# Lower extremity edema in patients with early ovarian cancer

**DOI:** 10.1186/1757-2215-7-28

**Published:** 2014-03-07

**Authors:** Myong Cheol Lim, Jeong Seon Lee, Byung Ho Nam, Sang-Soo Seo, Sokbom Kang, Sang-Yoon Park

**Affiliations:** 1 Center for Uterine Cancer, Research Institute and Hospital, National Cancer Center, 323, Ilsan-ro, Ilsandong-gu, Goyang-si 410-769, Gyeonggi-do, Republic of Korea; 2 Gynecologic Cancer Branch, Research Institute and Hospital, National Cancer Center, 323, Ilsan-ro, Ilsandong-gu, Goyang-si 410-769, Gyeonggi-do, Republic of Korea; 3 Biostatistics Biometric Research Branch, Research Institute and Hospital, National Cancer Center, 323, Ilsan-ro, Ilsandong-gu, Goyang-si 410-769, Gyeonggi-do, Republic of Korea

**Keywords:** Ovarian cancer, Lymphedema, Lower extremity edema

## Abstract

**Background:**

The objective of this study was to investigate clinical manifestations of lower extremity edema (LEE) in early ovarian cancer.

**Methods:**

Patients with early ovarian cancer who underwent staging surgery between January 2001 and December 2010. Medical records for LEE and/or responses to the Gynecologic Cancer Lymphedema Questionnaire (GCLQ) were evaluated.

**Results:**

Patients had a median age of 46 years. Twenty-nine patients (40.8%) had past (13 patients, 44.8%) and/or current patient-reported LEE (16 patients, 55.2%). Symptoms reported on the GCLQ in over 20% of respondents were numbness, firmness/tightness, swelling, heaviness, limited movement of knee, and aching. GCLQ total symptoms score was significantly higher in patients with current LEE. Most of the LEE (25/29, 86.2%) developed within 12 months after surgery and LEE lasted more than 6 months in approximately two-thirds of the patients (18/29, 62.1%). Only half of the patients (52.1%) indicated knowledge of lymphedema: 86.2% of LEE patients and 28.6% of patients with no LEE.

**Conclusions:**

Although a significant proportion of patients with ovarian cancer have LEE after surgery, most are not aware of lymphedema until they develop. Education and analyses for LEE and lymphedema are needed in patients with ovarian cancer.

## Background

Ovarian cancer is continuously increasing and the mortality is high [1-3]. Current standard treatment is cytoreductive surgery including lymph node dissection (LND) and chemotherapy. When there is no visible and palpable tumor in the peritoneal cavity during surgery, systematic pelvic and paraaortic LND is performed for staging and debulking. For advanced bulky ovarian cancer, the high rate of recurrence, which affects approximately two-thirds of the patients, is a critical issue. On the other hand, quality of life is one of the important concerns for patients with early stage ovarian cancer.

Lower extremity edema (LEE) after surgical treatment is one of the most important problems for women with ovarian cancer. About 20% of ovarian cancer patients develop LEE [4]. However, previous studies suffered from critical limitations in terms of reproducibility because of the heterogeneous study cohort that included patients with cervical, uterine, and vulvar cancer. Clinical information, such as stage, was not included [4-6]. Records concerning LEE and lower leg lymphedema (LLL) have been inconsistent. Generally, LEE is reported by patients as a questionnaire response and LLL is for the clinical diagnosis.

We investigated the clinical manifestations of LEE and LLL in patients with early ovarian cancer and reviewed the literature of LEE and LLL in patients with ovarian cancer.

## Methods

After obtaining Institutional Review Board (National Cancer Center, Korea) approval (NCCNCS-12-565), we reviewed medical records of patients with early ovarian cancer (FIGO stage I and II) at National Cancer Center who underwent cytoreductive and staging surgery between January 2001 and December 2010. Inclusion criteria were early stage epithelial ovarian cancer, no active treatment, available telephone communication with patients, and ability and willingness to provide verbal informed consent.

Medical records were reviewed. All patients who met the inclusion criteria were contacted by a telephone call by a investigator (JS LEE). The telephone interview questionnaire for LEE and gynecologic cancer lymphedema questionnaire (GCLQ) took approximately 20–30 min to complete. The questionnaire for LEE included onset, severity, location, duration, and management. Patients were also questioned concerning deep vein thrombosis to exclude other causes of LEE. LEE was defined as subjective edema of lower extremity based on patients’ complaint. LLL was defined based on a clinical diagnosis of lymphedema by a physician.

A previously developed GCLQ was pilot tested with patients with gynecologic cancers [7]. The GCLQ is satisfactory to distinguish patients with and without LEE [area under the curve (AUC), 0.95] and is easy to use. The patient-self-reported symptom scores, GCLQ, included seven symptom clusters: heaviness (item 14), swelling (general; items 8, 9, 20), swelling (limb; items 18, 19), infection-related (items 10 [redness], 11 [blistering], 13 [increased temperature in leg), aching (item 17), numbness (items 7, 12, 15, 16), and physical functioning (items 1–6) [7]. In the current study, Korean version of GCLQ (GCLQ-K) which was developed after minimal modifications from original GCLQ by our research team and showed high internal consistency and reproducibility was used [8]. In addition, ever or current LEE, location and onset of LEE, and any symptoms related to LEE were evaluated. Past LEE was an experience of LEE during a certain period and no current LEE. Current LEE was the existence of LEE at the time of survey irrespective of the onset of LEE. Ever LEE included the entire past and current LEE.

## Results

**Figure 1:**
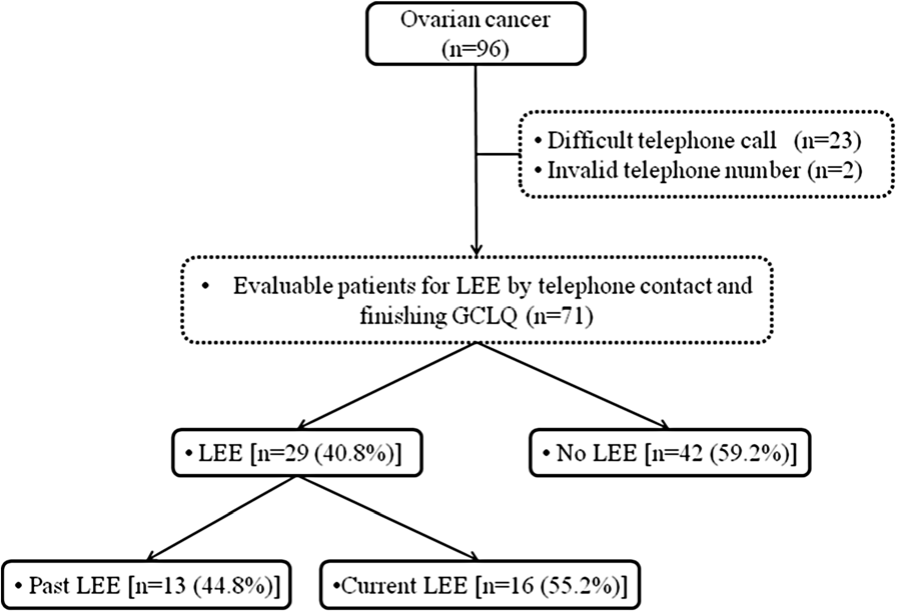
Flowchart for identifying patient-reported lower extremity edema in early ovarian cancer.

Of 96 patients with early epithelial ovarian cancer, 71 patients were contacted by telephone and their medical records were available (Figure 1). Characteristics of evaluable patients for LEE (n = 71) including age, body mass index, type of disease, FIGO stage, histology, CA125, and LN dissection are presented in Table 1. Most patients had ovarian cancer (n = 69) and the remaining two patients had tubal cancer. Fifty-two and 19 patients were identified and comprised the current FIGO stage I and II groups, respectively. The median age of the patients was 46 years (range, 22–65 years). Fifteen patients (21.1%) had serous histology. The remaining 56 patients (78.9%) had non-serous histology: mucinous (n = 11, 15.5%), endometrioid (n = 14, 19.7%), clear cell carcinoma (n = 15, 21.1%), transitional cell (n = 3, 4.2%), and other including mixed carcinoma (n = 13, 18.3%). Serum CA-125 was checked preoperatively in 68 patients (95.8%); 39 patients (57.4%) had elevated serum CA-125 level (≥35 U/mL). The median value of serum CA-125 was 42 U/mL (range, 1.9-4389 U/mL). Sixty-one patients (85.9%) received chemotherapy, which consisted of paclitaxel and carboplatin in 39 patients, paclitaxel and cisplatin in 20 patients, and cyclophosphamide and cisplatin (+Adriamycin) in two patients.

**Table 1:** Patients characteristics in early ovarian cancer (n = 71)

**Characteristics**	**Total**
Age (year) at time of operation	
Median (range)	46 (22–65)
Body weight (kg)	55.6 (42–78)
Height (cm)	158 (144–165)
Body mass index	23 (15.6-34.6)
Type of disease, n (%)	
Ovarian cancer	69 (97.2%)
Tubal cancer	2 (2.8%)
Stage (FIGO, 1989), n (%)	
Ia	25 (35.2%)
Ib	1 (1.4%)
Ic	26 (36.6%)
IIa	1 (1.4%)
IIb	10 (14.1%)
IIc	8 (11.3%)
Histology, n (%)	
Serous	15 (21.1%)
Mucinous	11 (15.5%)
Endometrioid	14 (19.7%)
Clear cell	15 (21.1%)
Transitional cell	3 (4.2%)
Others including mixed	13 (18.3%)
CA125 (U/mL)*	
Median (range)	42 (1.9–4389)
≥35, n (%)	39 (57.4%)
LN dissection, n (%)	69 (97.2%)
Number of LN dissected	
Median (range)	22 (3–98)
Chemotherapy	61 (85.9%)

FIGO, International Federation of Gynecology and Obstetrics; LN, lymph node.

*CA125 was available in 68 women.

**Figure 2:**
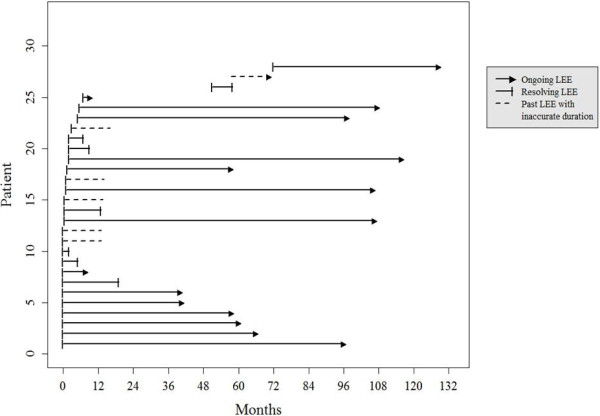
**Onset and duration of lower extremity edema in early ovarian cancer.** One line is omitted because of obscure onset and duration in one patient.

Of 71 evaluable patients, 29 (40.8%) patients had or previously had LEE. Of 29 patients, 16 patients (55.2%) had current LEE and 13 patients (44.8%) had past LEE. Clinical LLL was diagnosed in nine patients with ovarian cancer. All nine patients with a clinical diagnosis of LLL were included in the group of patients with LEE. One patient did not remember the exact onset time and end-point of LEE. Onset time and end-point of LEE could not be precisely identified in one and five patients, respectively. Accordingly, the duration of LEE was not definitive in seven patients. Figure 2 depicts the onset and duration of LEE. In the 27 patients with a clear onset of LEE, LEE occurred within 1 month after LND in 17 (63%), within 3 months after LND in five (18.5%), within 6 months after LND in two (7.4%), and within 12 months after LND in one (3.7%). In two patients, LEE developed 51 and 72 months after LND. More than half of the patients (16/29, 55.2%) had LEE at time of completing the questionnaire. In the 22 patients with a clear duration of LEE, the duration of LEE was within 6 months in six (27.3%), 12 month in two (9.1%), 60 months in five (22.7%), and more than 60 months in nine (40.9%).

Table 2 summarizes the patient-reported GCLQ data measured as “present with the past 4 weeks”. Symptoms in order of frequency were numbness (40.8%), firmness/tightness (22.5%), swelling (22.5%), heaviness (22.5%), limited movement of knee (21.1%), aching (21.1%), leg or foot feel weakness (18.3%), stiffness (15.5%), increased temperature in the leg (12.7%), limited movement of ankle (11.3%), and limited movement of foot (11.3%). The GCLQ score was significantly higher in patients with ever LEE compared to no LEE (*p* < 0.01).

**Table 2:** Frequency on the Gynecologic Cancer Lymphedema Questionnaire (GCLQ) items in early ovarian cancer (n = 71)

**Variables**							
**GCLQ items**	**SC**	**GCLQ lower extremity lymphedema symptoms items**	**Total n (%)**	**Past LEE n (%)**	**Current LEE n (%)**	**Ever LEE n (%)**	**No LEE n (%)**
15	N	Experienced numbness	29 (40.8%)	4 (5.6%)	8 (11.3%)	12(16.9%)	17 (23.9%)
12	N	Experienced firmness/tightness	16 (22.5%)	1 (1.4%)	8 (11.3%)	9 (12.7%)	7 (9.9%)
8	SW	Experienced swelling	16 (22.5%)	2 (2.8%)	12 (16.9%)	14(19.7%)	2 (2.8%)
14	H	Experienced heaviness	16 (22.5%)	2 (2.8%)	7 (9.9%)	9 (12.7%)	7 (9.9%)
2	PF	Limited movement of your knee	15 (21.1%)	2 (2.8%)	4 (5.6%)	6 (8.5%)	9 (12.7%)
17	A	Experienced aching	15 (21.1%)	0 (0.0%)	7 (9.9%)	7 (9.9%)	8 (11.3%)
6	PF	Leg or foot feel weak	13 (18.3%)	2 (2.8%)	4 (5.6%)	6 (8.5%)	7 (9.9%)
16	N	Experienced stiffness	11 (15.5%)	1 (1.4%)	3 (4.2%)	4 (5.6%)	7 (9.9%)
13	INF	Experienced increased temperature in the leg	9 (12.7%)	0 (0.0%)	3 (4.2%)	3 (4.2%)	6 (8.5%)
3	PF	Limited movement of your ankle	8 (11.3%)	1 (1.4%)	3 (4.2%)	4 (5.6%)	4 (5.6%)
4	PF	Limited movement of your foot	8 (11.3%)	1 (1.4%)	4 (5.6%)	5 (7.0%)	3 (4.2%)
19	LSW	Experienced groin swelling	6 (8.5%)	0 (0.0%)	4 (5.6%)	4 (5.6%)	2 (2.8%)
1	PF	Limited movement of your hip	5 (7.0%)	1 (1.4%)	0 (0.0%)	1 (1.4%)	4 (5.6%)
5	PF	Limited movement of your toe	5 (7.0%)	1 (1.4%)	2 (2.8%)	3 (4.2%)	2 (2.8%)
10	INF	Experienced redness	4 (5.6%)	1 (1.4%)	2 (2.8%)	3 (4.2%)	1 (1.4%)
7	N	Experienced tenderness	3 (4.2%)	0 (0.0%)	3 (18.8%)	3 (4.2%)	0 (0.0%)
9	SW	Experienced swelling with pitting	3 (4.2%)	1 (1.4%)	2 (2.8%)	3 (4.2%)	0 (0.0%)
18	LSW	Experienced hip swelling	3 (4.2%)	0 (0.0%)	1 (1.4%)	1 (1.4%)	2 (2.8%)
11	INF	Experienced blistering	2 (2.8%)	0 (0.0%)	0 (0.0%)	0 (0.0%)	2 (2.8%)
20	SW	Experienced pockets of fluid	0 (0.0%)	0 (0.0%)	0 (0.0%)	0 (0.0%)	0 (0.0%)

A, ache; H, heaviness; INF, infection; LEE, lower extremity edema; LLL, lower leg lymphedema; LSW, limb swelling; N, neuropathy; n, number; PF, physical function; SC, symptoms cluster; SW, swelling general.

Of the 29 patients with ever LEE, 10 (34.5%) patients made efforts to reduce the LEE by leg elevation using a pillow (n = 8), remedial exercise (n = 3), and other personalized exercise (n = 1). Of the 71 patients with early ovarian cancer and LND, only 52.1% (37/71) of the patients replied that they had general knowledge for LLL: 86.2% (25/29) and 28.6% (12/42) in patients with ever LEE and no LEE, respectively.

## Discussion

This is the first report to our knowledge that specifically addresses the incidence and duration of LEE in the ovarian cancer. The prevalence of ever LEE was 40.8, past was 18.3, and current was 22.5%. Most of the LEE (86.2%, 25/29) developed within 12 months after surgery and LEE lasted more than 6 months in approximately two-thirds of the patients (62.1%, 18/29).

The onset of LEE is consistent with previous results [6,9]. LEE and LLL are an important problem for ovarian cancer patients, because the current standard surgical treatment of ovarian cancer include lymph node dissection [10]. However, the real clinical impact of LEE and LLL on quality of life has not been specifically investigated (Table 3). We routinely offer education concerning the risk of lymphedema, lymphocyst, and dermatolymphangitis, and guidelines to prevent LLL in our routine clinical practice. More recently, we instituted a policy where all patients scheduled to undergo LND watch a video of exercise for lymphedema before surgery. However, only half of the patients with early ovarian cancer responded that they are aware of LLL. It seems that knowledge for lymphedema might be acquired post-LEE, considering the appreciable difference of knowledge depending on the existence of LEE (86.2% vs. 28.6%). Action and study for effective health care provider-patient communication is needed to narrow the gap between health care provider efforts and patient knowledge [11].

**Table 3:** Incidence and risk factors of edema and/or lymphedema of lower leg after treatment of ovarian cancer

**First author**	**Year**	**Study design**	**N. of OC patients (N. of total GC patients)**	**Stage**	**Diagnostic criteria**	**Incidence (%)**	**Risk factor for LEE/LLL**	**Comment**
Ryan M.	2003	CQ & MRR	141 (487)	NA	· Diagnosed LLL	· 7.1% (10/141) in OC	–	· MTFOTL: 3, 6, 12, and 60 months in 53, 18, 13, and 16% of the patients with GC.
						· 18.3% (89/487) in all GC		
						· 62.2% (28/45) after GLND		· Highest rate of LLL after GLND (50–62.2%).
						· 50.0% (47/233) after GLND + PLND		
Panici PB.	2005	Multi-center Italian RCT	427	III, 406 (95.1%)	· Diagnosed LLL	· 6.5% (14/216) vs. 0% (0/211) in SL vs. LNS	· SL compared to LNS	· Improvement of SL on PFS, but not OS.
				IV, 21 (4.9%)				
Magginoi A.	2006	Multi-center Italian RCT	268	I, 192 (72.7%)	· Diagnosed LLL	· 5.8% (8/138) vs. 0% (0/130) in SL vs. LNS	· SL compared to LNS	· No improvement of SL on PFS and OS.
				II, 72 (27.3%)				
Beesley V.	2007	PRO via mail	234 (802)	NA	· PRO - LEE	· LEE, 15.8% (37/234)	–	· Lowest incidence (4.7%) of LLL among GC
					· Diagnosed LLL			
						· LLL, 4.7% (11/234)		· BMI is not risk factor.
Tanaka T.	2007	CQ & MRR	21 (184)	I–II, 17 (81%)	· PRO - LEE	· 41.7% (5/12) in RC vs.	–	· RC is not risk factor. This should be investigated again in larger number of patients.
				III–IV, 4 (19%)		· 22.2% (2/9) in non-RC		
Tada H.	2009	Multi-center Japanese Retrospective	135 (694)	I–II, 75 (55.6%)	· Diagnosed & symptomatic LLL	· 20.7% (28/135)	· RT, OR 1.79 (95%CI, 1.20-2.68)	· MTFOTL: 4.6 (0.1–40.2) months
								· LLL, 25.8 vs. 31.7% in PALND(-) vs. (+)
				III–IV, 60 (44.4%)				
Matsuo K.	2011	Retrospective	276	I–II, 43 (15.6%)	· MRR	· LEE, 6.5% (18/276)	–	· LEE at initial diagnosis is an important on PFS (4.9 vs. 15.3 months) and OS (5.9 and 49.1 months).
				III–IV, 233 (84.5%)				
								· LEE is the 14th symptoms.
Karlan BY.	2012	RCT	161	Recurrent OC	· Peripheral edema	· LEE, 51-71% vs. 22% in AMG 386* vs. Control	AMG386 administered patients	· LEE, 51 & 71% (AMG 386 3 & 10 mg/kg QW with paclitaxel QW) vs. 22% in AMG 386* vs. Control (weekly paclitaxel (80 mg/m2 QW)
Achouri A.	2012	Retrospective	36 (88)	NA	· Diagnosed LLL	· 5.6% (2/36)	· Postoperative drainage, OR 0.13 (95%CI, 0.02-0.69)	· Incidence of LLL, 11.4% and 23.5% in EC and CC.
								· BMI, surgical approach (laparoscopy and laparotomy), PALND, SPOL, number of LND is not risk factor for LLL
Lim MC.	2013 Current study	CQ & MRR	71	I, 52 (73.3%)	· PRO - LEE	· 40.8% (29/71)	–	· MTFOTL: <1, 3, 6, and 12 months in 63,18.5, 7.4, and 3.7%
				II, 19 (26.7%)				
								· Median duration of LEE: <6, 12, 60, and ≥60 in 27.3, 9.1, 22.7, and 40.9%

BMI, body mass index; CC, cervical cancer; CQ, cross-sectional questionnaire; EC, endometrial cancer; GC, gynecologic cancer; GLND, groin lymph node dissection; LEE, lower extremity edema; LLL, lower leg lymphedema; LND, lymph node dissection; LNS, lymph node sampling; MRR, medical record review; MTFOTL, median time from operation to lower extremity edema; N, number; NA, not available; OC, ovarian cancer; OS, overall survival; PALN, paraaortic lymph node; PALND, paraaortic lymph node dissection; PFS, progression free survival; PLN, pelvic lymph node; PLND, pelvic lymph node dissection; PRO, patient reported outcomes; QW, once weekly; RC, retroperitoneal closure; RCT, randomized trial; RT, radiotherapy; SL, systemic pelvic and aortic lymph node dissection; SPOL, symptomatic postoperative lymphocele.

*AMG386, an investigational peptide-Fc fusion protein that neutralizes the interaction between the Tie2 receptor and angiopoietin-1/2.

From two randomized trials of LND, the risk of LLL significantly increased in a group with systematic LND compared to a group with lymph node sampling in early (5.8% vs. 0%) and advanced (6.5% vs. 0%) ovarian cancer [12,13]. The site of the lymph node dissected is an important factor related to LEE/LLL. Paraaortic LND (25.8 vs. 31.7%, *p* = 0.158) does not increase the risk of LLL [9]. The prevalence of LEE was significantly elevated after LND including the groin: 18.0% for pelvis and paraaortic; 20.2% for pelvis only; 50% for pelvis and groin, and 62.2% for groin only [6]. Groin and pelvis is the critical area for LEE/LLL. Paraaortic LND is not a risk factor [5,6]. In this study, most of women with early ovarian cancer underwent systematic LND in the pelvis and paraaortic area. Retroperitoneal closure was investigated and found not to be a risk factor of LEE despite a significant numerical difference (41.7% vs. 22.2%) [14]. Because of the small number of patients (n = 21), further studies are needed to confirm this. In this study, only the peritoneum of the aortic area was closed in patients with ovarian cancer. The exact role of retroperitoneal closure should be confirmed in a large patient cohort. Body mass index, surgical approach (laparotomy vs. laparoscopy), and symptomatic postoperative lymphocele are not risk factors of LEE/LLL [4,5].

Two randomized trials analyzed survival for systematic LND in ovarian cancer [12,13]. In one study, systemic LND improved only progress-free survival in advanced ovarian cancer [13]. In early ovarian cancer, there is no survival benefit from systematic LND [12]. The power was 80% implying insufficient power to exclude clinically important effects of systematic LND on survival [12]. LND is still one of the standard staging and cytoreductive surgical procedures. At this point, gynecologic oncologists, medical oncologists who perform adjuvant chemotherapy, and patients with ovarian cancer should be aware of the pattern of LEE/LEE [10]. Figure 2 illustrates the pattern of LEE after cytoreductive surgery and adjuvant chemotherapy, in close agreement with previous studies [6,9]. We first investigated the duration of LEE (Figure 2). This information should be discussed with patients before LND.

On the other hand, Matsuo et al. reported that LEE at time of the initial diagnosis of ovarian cancer is a strong prognostic indicator of PFS (4.9 vs. 15.3 months, *p* < 0.01) and overall survival (5.9 and 49.1 months, *p* < 0.01) [15]. Previously, we reported that LEE as a clinical manifestation of deep vein thrombosis, suggesting disease burden, might be a poor prognostic marker in survival of ovarian cancer [16,17]. In the current study, 41.4% (12/29) of the patients responded that they had LEE immediately after surgery. However, the prognostic role of LEE is difficult to analyze because of the limited disease failure in early ovarian cancer. Theoretically, LEE could easily develop in patients with deep vein thrombosis from extensive disease and compression of lymphatic vessel from bulky lymph node metastasis. The prognostic role of LEE in ovarian cancer should be investigated.

The limitations of this study are that it was not a prospective study, that LEE/LLL related events like postoperative lymphocyst and/or dermatolymphangitis were not analyzed, and the lack of confirmation of the relationship of LEE and LLL because a significant portion of the patients in this study visited only annually. And there is a possibility of bias because this was a retrospective and cross-sectional study. The strengths of this study are the homogenous patient cohort in terms of stage, surgical principle, and adjuvant chemotherapy; the use of a validated questionnaire [7]; and the clear description of the duration of LEE.

## Conclusions

In the current study, significant numbers of patients with ovarian cancer have LEE after primary treatment of ovarian cancer. However, most of them were unfamiliar with lymphedema until they actually develop LEE. Therefore, preoperative and postoperative counseling and education for prevention and early sign of LEE/LLL should be provided to the patients who will undergo LND. And prospective studies for effective educational interventions on LEE/LLL are needed in patients with ovarian cancer.

## Abbreviations

FIGO: International Federation of Gynecology and Obstetrics; LEE: Lower extremity edema; LLL: Lower leg lymphedema; LND: Lymph node dissection.

## Competing interests

No potential competing interest are disclosed.

## Authors’ contributions

MCL and SYP managed the overall project. All authors participated in research design and contributed to the writing and revising of the manuscript. JSL surveyed and collected all data. BHN performed primary analysis of the data. All authors read and approved the final manuscript.
